# Isobaric Thermal Expansivity and Isothermal Compressibility of Liquid Metals

**DOI:** 10.3390/ma16103801

**Published:** 2023-05-17

**Authors:** Yuri N. Starodubtsev, Vladimir S. Tsepelev

**Affiliations:** 1 Research Center for Physics of Metal Liquids, Ural Federal University, Yekaterinburg 620002, Russia; 2 Gammamet Research and Production Enterprise, Yekaterinburg 620131, Russia

**Keywords:** isobaric thermal expansivity, isothermal compressibility, thermal pressure, internal pressure, Grüneisen parameter, liquid metals, melting point

## Abstract

The relationship between the volumetric thermodynamic coefficients of liquid metals at the melting point and interatomic bond energy was studied. Using dimensional analysis, we obtained equations that connect cohesive energy with thermodynamic coefficients. The relationships were confirmed by experimental data for alkali, alkaline earth, rare earth, and transition metals. Cohesive energy is proportional to the square root of the ratio of melting point *T_m_* divided by thermal expansivity α*_p_*. Thermal expansivity does not depend on the atomic size and atomic vibration amplitude. Bulk compressibility β*_T_* and internal pressure *p_i_* are related to the atomic vibration amplitude by an exponential dependence. Thermal pressure *p_th_* decreases with an increasing atomic size. Fcc and hcp metals with high packing density, as well as alkali metals, have the relationships with the highest coefficient of determination. The contribution of electrons and atomic vibrations to the Grüneisen parameter can be calculated for liquid metals at their melting point.

## 1. Introduction

Metal melting is the initial stage of most technological processes. Therefore, research on metallic liquids is of great practical importance. The simplest objects are monoatomic liquids. In the model of a simple liquid, atoms do not have dipole moments, are not chemically bound, and have a spherically symmetric interaction potential [1,2]. In the physical lattice model, it is assumed that the liquid has a lattice structure, at least in the first coordination sphere [3], and the atomic oscillation occurs inside the free volume, i.e., the space available for the movement of the atom [4].

The molecular kinetic theory also provides relationships that are adequately related to the experimental data. Lindemann [5] calculated the vibration frequency of atoms at the melting point. Using the frequency of atomic vibrations, Andrade [6] derived a formula that relates dynamic viscosity to molar mass, volume and melting point.

In the Einstein model, the substance contains a large number of independent quantum harmonic oscillators of the same natural atomic frequency, and above the Einstein temperature, the heat capacity increases slightly [7]. In the Debye model, quantum harmonic oscillators have different frequencies, and above the Debye temperature Θ*_D_*, all modes of atomic vibrations are excited. In the high temperature approximation, the Einstein and Debye temperatures differ slightly [8]. Grimvall and Sjödin [9] investigated the relationship between the different physical properties of materials and the Debye temperature. In [10], the analysis of the properties of metallic liquid is considered in detail.

In this work, we will investigate the thermodynamic coefficients that characterize the change in the volume of monatomic liquid metals. The isobaric thermal expansivity α*_p_* determines the temperature change of volume *V* at constant pressure *p*. Thermal expansion is associated with the asymmetry of interatomic interaction [11,12], and as a result, the repulsive force grows faster than the attractive force. Upon transition to the liquid state, bulk thermal expansion sharply increases [13]. The isothermal compressibility β*_T_* determines the change in volume under pressure at a constant temperature *T*. Isobaric thermal expansivity and isothermal compressibility are directly related to the thermal pressure *p_th_*, the internal pressure *p_i_*, and the Grüneisen parameter [14].

Molar mass and volume, as well as the interatomic bond energy, represent the most general atomic characteristic of a substance. Interatomic interaction characterizes the cohesion energy, which is equal to the energy required for the decomposition of a solid into independent atoms at 0 K. Thermal energy is used to compare the strength of an interatomic interaction with the destructive effect of thermal motion. It was demonstrated that isobaric thermal expansivity [15,16,17], bulk modulus [9,18], and internal pressure [19,20] are related to interatomic bond energy. In particular, the isobaric thermal expansivity is inversely proportional to cohesion energy [16] and melting point [21].

This article describes new data on the relationship between volumetric thermodynamic coefficients with atomic characteristics and the interatomic interaction in liquid metals at their melting point.

## 2. Method

We used dimensional analysis to identify the relationship between physical and chemical quantities [22,23]. Each object is characterized by a set of significant physical quantities. At the first stage, we identified these quantities. Then, we found relations between significant quantities that provided the same dimension on the left and right side of the equation. The dimensional analysis provided good results in the study on the viscosity, surface tension, and self-diffusion of metallic liquids [24,25,26]. The adjusted coefficient of determination *R*^2^*_adj_* was used to assess how well a relationship predicts outcomes. The better the linear regression fits the data in comparison to the simple average, the closer the value of *R*^2^*_adj_* is to 1.

The melting point at which the metal passes from the solid to the liquid state was chosen as the characteristic temperature. At this temperature, the short range order of liquid metals can be close to crystalline [27,28]. The quantities at the melting point are marked with subindex *m*. Data on the cohesion energy *E_c_* (J·mol^−1^), melting point *T_m_* (K) molar volume *V_m_* (m^3^·mol^−1^), heat capacity at constant pressure *C_p_* (J·mol^−1^·K^−1^) and atomic mass *m* (kg) were taken from [10]. Reference [29] contains the Debye temperature Θ*_D_* (K), and references [30,31] contain the Fermi energy *E_F_* (J·mol^−1^). Reference [32] contains the Wigner–Sietz radius *r_s_* (m) and the valency of chemical element *z*. The isothermal compressibility at melting point β*_Tm_* (J^−1^·m^3^) was taken from [30,33], and the isobaric thermal expansivity at melting point α*_pm_* (K^−1^) was from [10,33]. In this work, we analyzed data for alkali, alkaline earth, rare earth, and transition metals.

## 3. Results and Discussion

The dimension analysis of significant thermophysical quantities allowed us to obtain the simple relations for the thermal expansivity α*_p_* and the isothermal compressibility β*_T_*:

(1) $$E\propto R{\left(\frac{T}{\alpha_{p}}\right)}^{0.5},$$

(2) $$E\propto \frac{V}{\beta_{T}},$$

where *E* is the molar energy of the interatomic bond (J·mol^−1^), *V* is the molar volume (m^3^·mol^−1^), and *R* is the universal gas constant (J·K^−1^·mol^−1^).

**Figure 1:**
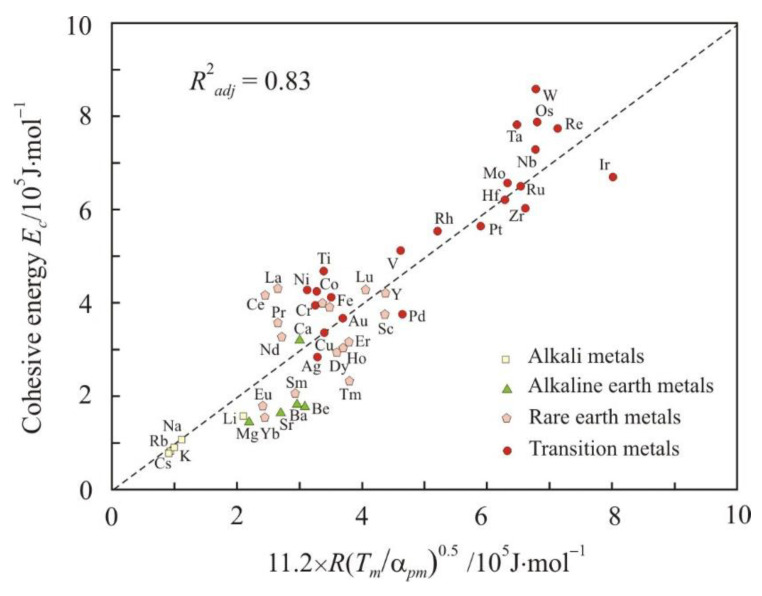
Relationship between the cohesive energy *E_c_* and 11.2 × *R*(*T_m_*/α*_pm_*)^0.5^ parameter. The dashed line corresponds to the equality *E_c_* = 11.2*R*(*T_m_*/α*_pm_*)^0.5^.

**Figure 2:**
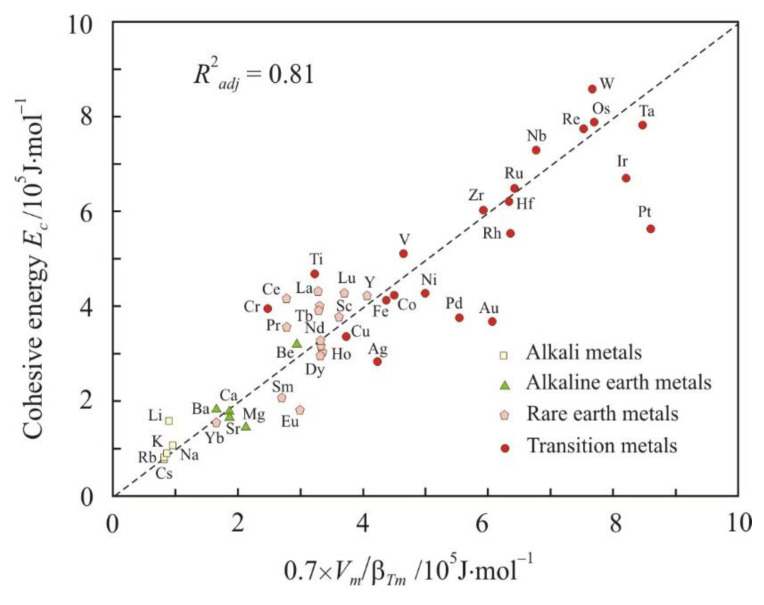
Relationship between the cohesive energy *E_c_* and 0.7 × *V_m_*/β*_Tm_* parameter. The dashed line corresponds to the equality *E_c_* = 0.7 × *V_m_*/β*_Tm_*.

Figure 1 and Figure 2 show relations (1) and (2) at the melting point *T_m_* in the form:

(3) $$E_{c}=11.2R{\left(\frac{T_{m}}{\alpha_{pm}}\right)}^{0.5},$$

(4) $$E_{c}=0.7\frac{V_{m}}{\beta_{Tm}}.$$

Here, the cohesive energy *E_c_* was used as the energy of the interatomic bond. Dimensionless factor 11.2 in formula (3) is equal to the average ratio of *E_c_* to *R*(*T_m_*/α*_pm_*)^0.5^ for all metals, and dimensionless factor 0.7 in formula (4) is equal to the average ratio of *E_c_* to *V_m_*/β*_Tm_*. For Equations (3) and (4), the coefficient of determination *R*^2^*_adj_* exceeds 0.80, and these equations can be assessed as good. Relation (3) between the cohesive energy and the thermal expansivity was obtained for the first time. Relation (4) is more obvious and has already been used in different versions [30,34].

The analysis shows that Equations (3) and (4) for alkali metals, with the exception of lithium, have a coefficient of determination greater than 0.95. In addition, a high coefficient of determination is observed in metals with face-centered cubic (fcc) and hexagonal closed-packed (hcp) lattices, which have the highest packing density, 74%. For example, the relationship between the cohesive energy and the *R*(*T_m_*/α*_pm_*)^0.5^ parameter has *R*^2^*_adj_* = 0.96 for fcc metals. Body-centered cubic (bcc) metals, with a lower packing density of 68%, show more scattering around Equations (3) and (4). The influence of the lattices type on the properties of metals in the liquid state can be associated with the preservation of the local atomic structure near the melting point [35].

The isochoric thermal pressure coefficient *p_th_* characterizes the change in the pressure with a change in temperature at a constant volume, and from relations (1) and (2), we reduce it to this form:

(5) $$p_{th}={\left(\frac{\partial p}{\partial T}\right)}_{V}=\frac{\alpha_{p}}{\beta_{T}}\propto \frac{R^{2}T}{EV}.$$

The internal pressure characterizes the change in the internal energy *U* (J) with a change in volume at a constant temperature, and at normal pressure, one can use this approximation [20]:

(6) $$p_{i}={\left(\frac{\partial U}{\partial V}\right)}_{T}=\frac{\alpha_{p}T}{\beta_{T}}-p\approx \frac{\alpha_{p}T}{\beta_{T}}\propto \frac{R^{2}T^{2}}{EV}.$$

If we take *E* = *RT_m_*, then relations (5) and (6) at the melting point can be reduced to this simple form:

(7) $$p_{th}{}_{m}\propto \frac{R}{V_{m}},$$

(8) $$p_{im}\propto \frac{RT_{m}}{V_{m}}.$$

**Figure 3:**
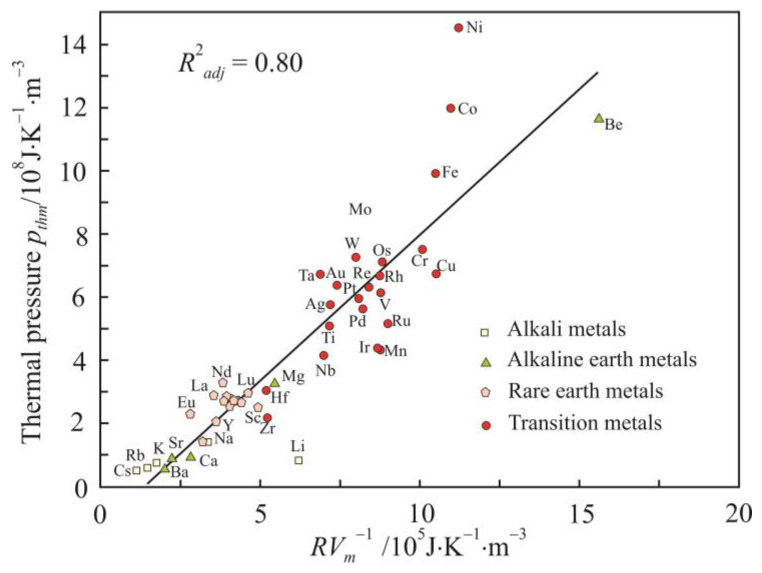
Relationship between the thermal pressure *p_thm_* and *RV_m_*^−1^ parameter.

**Figure 4:**
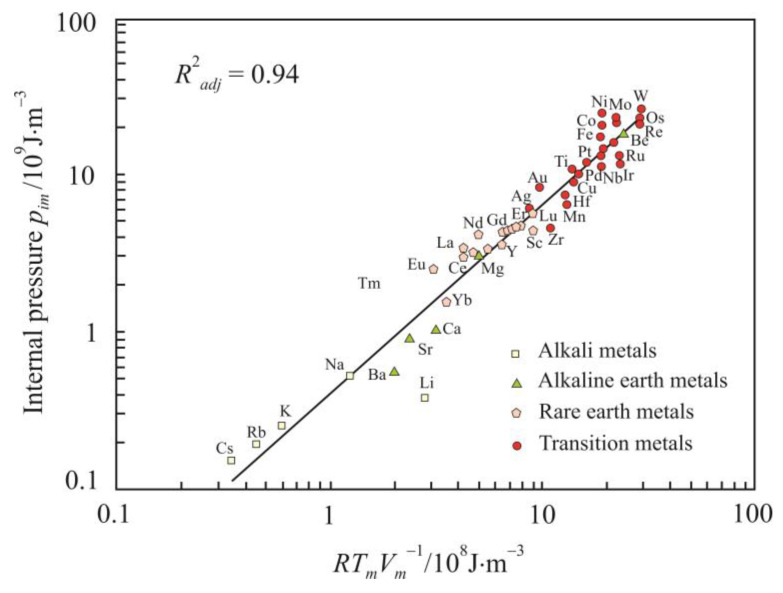
Relationship between the internal pressure *p_im_* and thermal energy density *RT_m_V_m_*^−1^.

Figure 3 and Figure 4 show relations (7) and (8) in the form of a linear regression. For clarity, relation (7) is presented on a logarithmic scale. The high coefficients of determination, especially for internal pressure *p_im_*, confirms the significance of initial relations (1) and (2) obtained using the dimensional analysis.

The thermal pressure *p_th_* is inversely proportional to the molar volume *V_m_* (Figure 3). The ferromagnetic metals Ni, Co, and Fe noticeably deviate from the linear regression. The molar volume is related to the atomic size *a* by the relation

(9) $$V_{m}=N_{A}a^{3},$$

where *N_A_* is the Avogadro constant (mol^−1^). So, the thermal pressure decreases with increasing atomic size. Thermal expansivity analysis showed that α*_pm_* does not depend on the atomic size and does not affect dependency (5). The independence is confirmed by the random distribution of points on the experimental dependence of α*_pm_* on the atomic size *a*.

Figure 4 demonstrates the strong relationship between internal pressure *p_im_* and thermal energy density *RT_m_V_m_*^−1^. This suggests that the internal pressure at the melting point is mainly due to the thermal vibration of atoms.

Relations (7) and (8) also show a higher coefficient of determination for fcc and hcp metals. For alkaline earth metals, the coefficient of determination exceeds 0.995, and for alkali metals, with the exception of lithium, it is above 0.999.

The amplitude of atomic vibrations *A_m_* (m) was determined from the kinetic theory [24]:

(10) $$A_{m}=\frac{h}{\pi \;\Theta_{D}}\sqrt{\frac{T_{m}}{2k_{B}m}},$$

where Θ*_D_* is the Debye temperature (K), *h* is the Plank constant (J·s), and *m* is the atomic mass (kg). It follows from (10) that the amplitude increases with an increase in the melting point *T_m_* and a decrease in the atomic mass *m* and Debye temperature Θ*_D_*.

**Figure 5:**
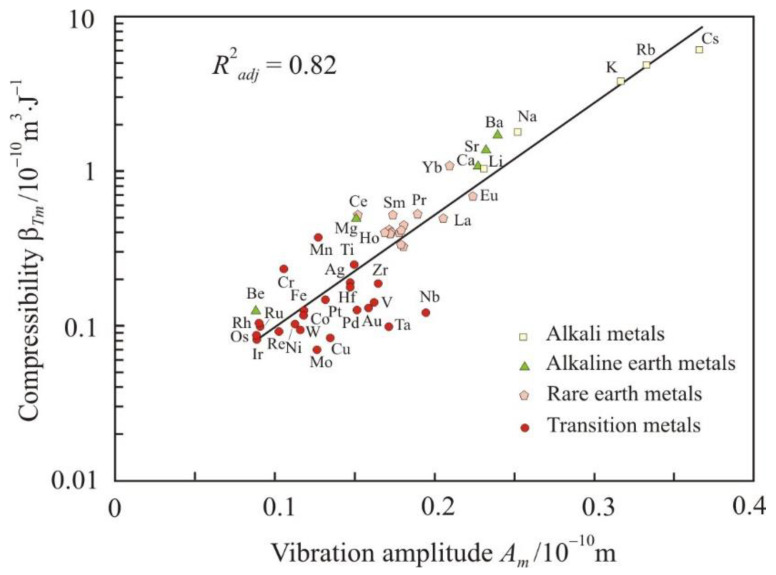
Dependence of the isothermal compressibility β*_Tm_* on the amplitude of atomic vibrations at the melting point *A_m_*.

**Figure 6:**
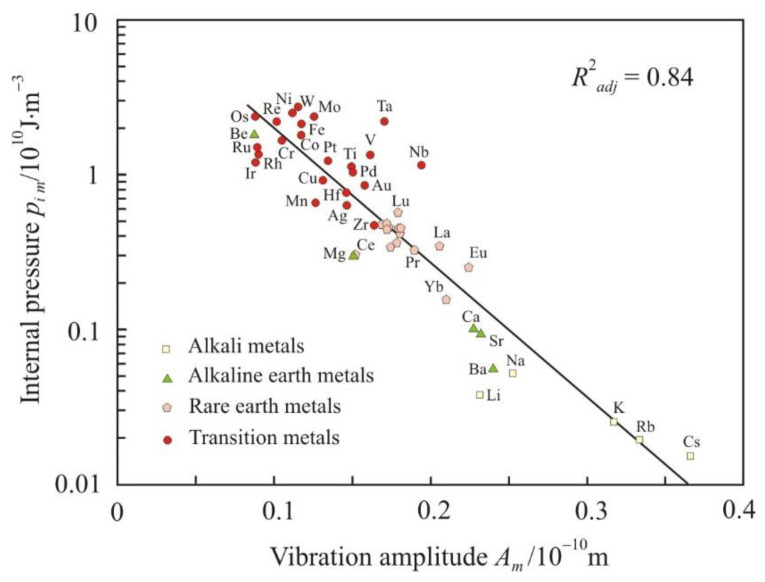
Dependence of the internal pressure *p_im_* on the amplitude of atomic vibrations at the melting point *A_m_*.

For strong interatomic bonds, the amplitude of atomic vibrations is smaller. Essentially, the amplitude also characterizes the interatomic interaction. Figure 5 and Figure 6 show the dependences of isothermal compressibility β*_Tm_* and internal pressure *p_im_* on the amplitude of atomic vibrations at the melting point. The compressibility increases and the internal pressure decreases when the amplitude increase. Dependencies are linear if β*_Tm_* and *p_im_* are presented on a logarithmic scale. Therefore, dependency can be written as an exponential:

(11) $$\beta_{Tm}=0.019e^{\;0.166A_{m}},$$

(12) $$p_{im}=14.4e^{\;0.2A_{m}}.$$

where the vibration amplitude is given in picometers (pm) and the isothermal compressibility β*_Tm_* and internal pressure *p_im_* are given in SI base units. The thermal expansivity α*_pm_* does not depend on the amplitude of atomic vibrations, and the thermal pressure *p_thm_* has a noticeably lower coefficient of determination of 0.67.

The relationship between the thermal expansivity α*_pm_* and the vibration amplitude *A_m_* is characterized by a coefficient of determination close to zero. This confirms the concept of anharmonicity [11,12], according to which the thermal expansion is related to the nonlinearity of the interatomic interaction force.

The thermal expansivity and isothermal compressibility are also included in the expression for the Grüneisen parameter [36]:

(13) $$\gamma =V{\left(\frac{\partial p}{\partial U}\right)}_{V}=\frac{\alpha_{p}V_{m}}{\beta_{T}C_{V}},$$

where *C_V_* (J·mol^−1^·K^−1^) is the heat capacity at constant volume. The Grüneisen parameter characterizes the isochoric change in the internal energy density *U*/*V* with a change in pressure.

In the Debye model, the parameter γ is related to the atomic vibration and is calculated using the formula

(14) $$\gamma_{vib}=-\frac{V}{\Theta_{D}}{\left(\frac{\partial \Theta_{D}}{\partial V}\right)}_{T}=-{\left(\frac{\partial \ln\Theta_{D}}{\partial \ln V}\right)}_{T}$$

**Figure 7:**
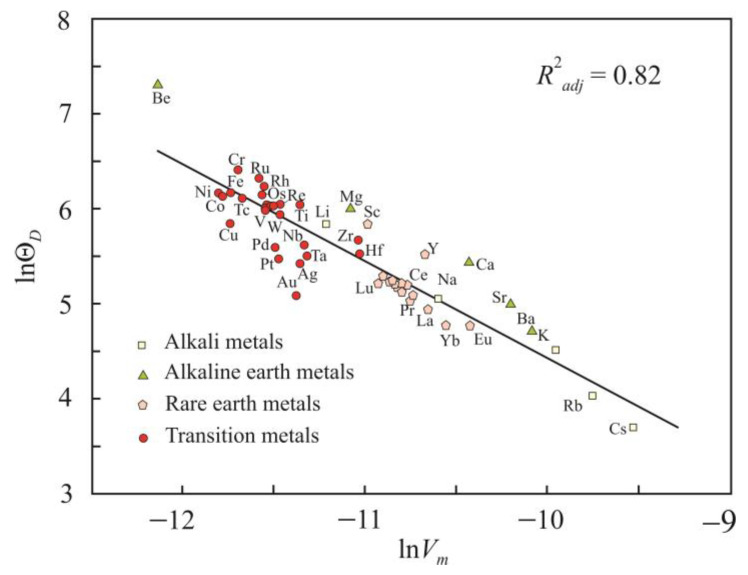
Dependence of the Debye temperature lnΘ*_D_* on the molar volume ln*V_m_* for liquid metals at the melting point.

We choose melting point *T_m_* as a fixed temperature. Figure 7 shows the dependence of lnΘ*_D_* on ln*V_m_*, from which γ*_vib_* = 1.02 can be found. Thus, the average vibrational component of the Grüneisen parameter of liquid metals at the melting point is 1.02.

The electronic contribution to the Grüneisen parameter [37,38] is

(15) $$\gamma_{el}=-{\left(\frac{\partial \ln D}{\partial \ln\rho }\right)}_{T},$$

where *D* (J^−1^) is the electronic density of states at the Fermi energy level, and ρ (m^–3^) is the concentration of free electrons. According to [7], the electron density of the state is

(16) $$D\propto E_{F}^{0.5}V_{m}.$$

The free electron density ρ can be calculated from the Wigner–Seitz radius *r_s_* [39]:

(17) $$\frac{1}{\rho }=\frac{4\pi \;r_{s}^{3}}{3}.$$

**Figure 8:**
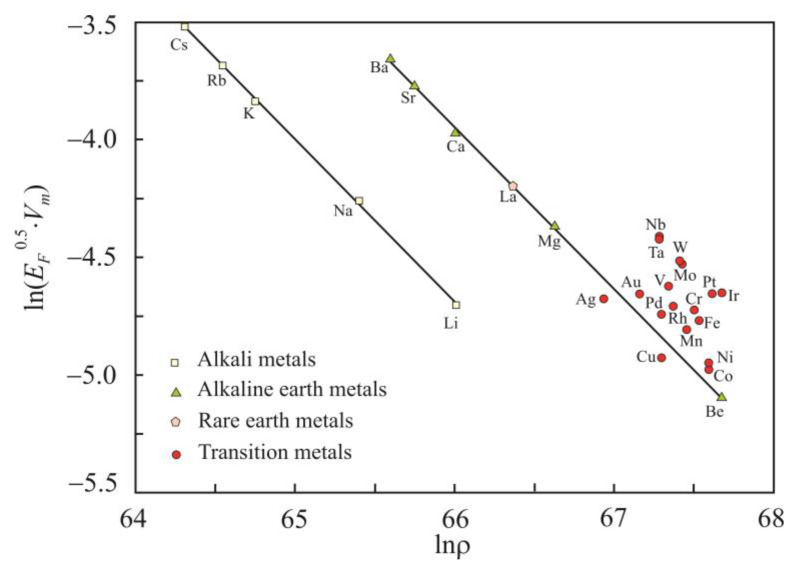
Dependence of ln(*E_F_*^0.5^*V_m_*) on the electron concentration lnρ for liquid metals at the melting point.

The Wigner–Seitz radius is the radius of a sphere whose volume is equal to the volume per a free electron. The concentration of free electrons can also be obtained from the formula

(18) $$\rho =\frac{z\;N_{A}}{V_{m}},$$

where *z* is the valency of chemical elements. Figure 8 shows the dependence of ln(*E_F_*^0.5^*V_m_*) on lnρ. It follows that for alkali and alkaline earth metals, there is a strong relationship, with a determination coefficient of 0.999, and the electronic contribution to the Grüneisen parameter is 0.60.

Dimensionless Grüneisen parameter γ is a useful quantity for characterizing the vibrational anharmonicity that causes thermal expansion. The expression for the difference in heat capacities (*C_p_* − *C_v_*) contains the square of the thermal expansivity [40]:

(19) $$C_{p}-C_{v}=\frac{\alpha_{p}^{2}VT}{\beta_{T}}.$$

**Figure 9:**
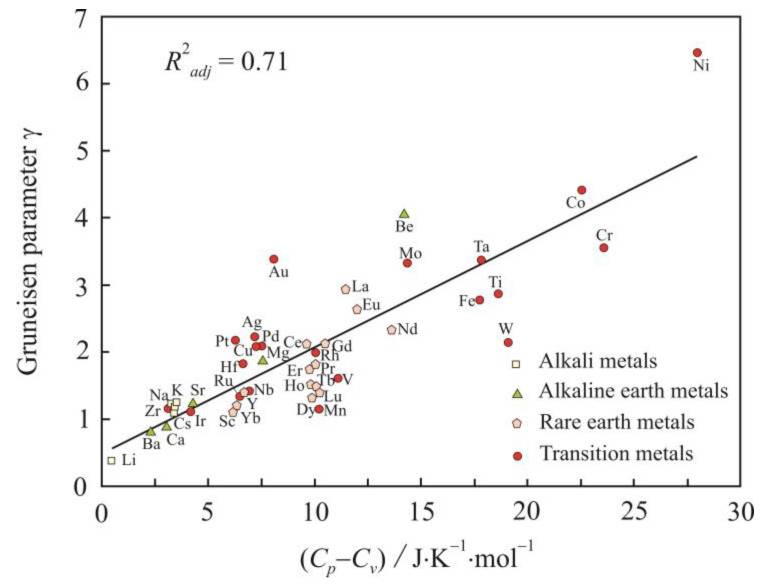
Relation of Grüneisen parameter γ to the difference in heat capacities (*C_p_* − *C_v_*) for liquid metals at the melting point.

Figure 9 shows a fairly close relationship between the Grüneisen parameter γ and the difference in heat capacities (*C_p_* − *C_v_*) for metals at the melting point, and it confirms the relationship between γ and vibrational anharmonicity.

## 4. Conclusions

The relationships between the volumetric thermodynamic coefficients of liquid metals at the melting point and the energy of interatomic interaction were investigated. The isobaric thermal expansivity α*_p_*, isothermal compressibility β*_T_*, isochoric thermal pressure *p_th_*, internal pressure *p_i_*, and the Grüneisen parameter were used as thermodynamic coefficients. The energy quantities were molar cohesive energy *E_c_* and thermal energy *RT_m_*. Using the dimensional analysis, we obtained equations that relate the cohesive energy to the thermodynamic coefficients. The relationships were confirmed with experimental data from alkali, alkaline earth, rare earth, transition metals.

The following results were obtained in this work: Thermodynamic coefficients at the melting point depend on the type of crystal lattice. Ratios for liquid metals with face-centered cubic and hexagonal closed-packed lattices as well as for alkali and alkaline earth metals have the highest coefficient of determination. Cohesive energy is proportional to the square root of the ratio of the melting point *T_m_* divided by thermal expansivity α*_p_*. Thermal expansivity α*_p_* does not depend on atomic size and atomic vibration amplitude. This confirms the concept of anharmonicity of thermal expansion. Bulk compressibility β*_T_* and internal pressure *p_i_* are related to atomic vibration amplitude by an exponential dependence. Internal pressure is closely related to thermal energy density, and at melting point, it is mainly due to the thermal vibration of the atoms. Thermal pressure *p_th_* decreases with an increasing atomic size. The contribution of electrons and atomic vibrations to the Grüneisen parameter γ can be calculated for liquid metals at the melting point.

## Data Availability

The data presented in this article are available upon request from the corresponding author.
